# Online Emotional Support Accompany Group Intervention and Emotional Change of the Public During the COVID-19 Pandemic: A Multi-Period Data Analysis From China

**DOI:** 10.3389/fpsyg.2022.840686

**Published:** 2022-04-28

**Authors:** Xiaohua Lu, Xinyuan Wang, Yingjun Zhang, Zheng Ma, Shixin Huo, Tao Bu, Daisheng Tang

**Affiliations:** ^1^Counseling Center, Beijing Jiaotong University, Beijing, China; ^2^School of Economics and Management, Beijing Jiaotong University, Beijing, China; ^3^Mental Health Education and Counseling Center, Beijing Normal University, Beijing, China

**Keywords:** COVID-19, social distancing, emotional distress, online emotional support accompany group, psychological crisis intervention

## Abstract

COVID-19 has made it difficult to adopt traditional face-to-face psychological intervention under this situation because of the blocked down and social distancing, which brings big psychological crisis to the public among the global. To explore the emotional change of the public in China at the outburst of the pandemic at different phases, to establish an online working platform and create a new model of an online intervention to hold public emotions under pandemic, and test its effectiveness, so to give advisement for government emergency management system. We established an online organization to work for this program ad innovated a model of online group counseling with online emotional support accompany group (OESAG) right after the outburst of a pandemic. We analyzed 53 OESAGs from February 10 to April 9, including 555 application forms, 253 feedback from members, and 139 feedback from group leaders by using NVivo and SPSS to explore the evolution and characteristics of public emotion during COVID-19 and the effectiveness of OESAG. Our results showed that the emotional changes of members ranged from shock to depression to positive. The public's emotions swiftly changed from stress, anxiety, and isolation, to the hope of returning to work or finding a job during the pandemic with the help of OESAG. OESAG has effectively regulated the negative emotions of members by conducting psychological crisis intervention to provide members a space to communicate with each other, especially the female and frontline staff. Policy makers can set up an online systematic psychological crisis intervention system as soon as possible to make up for the lack of psychological assistance in the emergency management system.

## Introduction

The corona virus disease 2019 (COVID-19) pandemic was a big and quick-hit that affected almost every aspect of the daily life of the public. While a lot of people lost their lives, others have been quarantined and kept a social distance from others. Many people have thus reported feelings of rejection, isolation, panic, and depression (Naqvi, 2020; Taylor et al., 2020b), and some have even committed suicide (Elbogen et al., 2020). These are all causes of mental health problems (Li et al., 2021; Xu et al., 2021), psychological crisis (Abir et al., 2021; Pérès et al., 2021), and even PTSD (Budden, 2009; Fenster et al., 2018), which may, in turn, bring an unstable society and potential decrease in productivity. Psychotherapy is very important under this pandemic, but it is also difficult to perform traditional in-person psychological interventions. Also, individual psychotherapy fails to meet the huge demand. Thus, a model of online group psychotherapy needs to be innovated.

There is evidence of widespread emotional distress in response to the COVID-19 pandemic. On February 10, February 18, and March 8, 2020, the number of people infected with the COVID-19 pandemic in China was 37,626, 74,185, and 80,735, and the cumulative number of deaths was 1,016, 2,004, and 3,119, respectively. Although the increase in the number of infections has declined, the cumulative deaths are still increasing, and this may cause a psychological crisis for the Chinese public. Data from China, for example, suggests that 25% of the general population has experienced moderate to severe levels of anxiety- or stress-related symptoms in response to COVID-19 (Qiu et al., 2020; Wang et al., 2020). Likewise, there is evidence of considerable distress specific to COVID-19. Indeed, several investigators have reported elevated levels of fear of infection (Ahorsu et al., 2020; Lee and Neimeyer, 2020; Mertens et al., 2020), as well as an elevated prevalence of posttraumatic stress disorder (Tan et al., 2020). Recent research based on data collected in the early stages of the COVID-19 epidemic from a large American and Canadian population-representative sample suggests that epidemic-related distress may comprise a network of five interconnected symptom categories—danger and contamination fears, socioeconomic concerns, xenophobia, traumatic stress symptoms, and compulsive checking and reassurance seeking—corresponding to a COVID stress syndrome (Taylor et al., 2020a,b).

In response to the psychological crisis brought by the COVID-19 pandemic to the public, there have been some psychological crisis intervention studies (Brouzos et al., 2021). However, there is no social-psychological service guarantee mechanism in the emergency management system of China. The emergency management life cycle can be divided into four parts: disaster prevention and mitigation, emergency preparedness, emergency response, recovery, and reconstruction. Emergency response has attracted the attention of the government and the public (Kong and Sun, 2021). As an important part of emergency response, psychological intervention and assistance have not received enough attention in China. This is not conducive to the recovery of the public psychological crisis during and after the pandemic.

Therefore, we have designed the online emotional support accompany Group (OESAG) to make up for the lack of psychological intervention and psychological assistance in the emergency management system of China. By providing virtual space for members who are our psychological crisis intervention object to share their emotions, they are able to see and be seen by themselves and others to support each other and get feedback to make them feel connected, supported, safe, and secure, and they can be empowered to endure the vulnerability under pandemic. OESAG was created right after the pandemic and got provided to the public during the pandemic; emotional data before and after the group psychotherapy was also got collected to describe the emotional change of the public and intervene in emotional distress during the pandemic. Furthermore, we would improve the effectiveness of OESAG in psychological crisis intervention during COVID-19 by using the triangulation method which combines qualitative analysis, quantitative analysis, and case analysis.

## Literature Review

### Emotion and Psychological Crisis

There is no single definition of emotion. According to Oxford English Dictionary, emotion is a mental state (Michel, 2002). Emotion differs in the positive–negative dimension and can be divided into different categories of emotions such as interest, enjoyment, sadness, anger, fear, etc (Izard, 2009; Izard et al., 2011). In order to adapt to changes in the environment, people's emotions are constantly changing, and when stimulated by changes in the external environment, people's emotions are activated (Levenson, 1999). COVID-19 is a public health emergency of international concern that has caused a huge psychological crisis (Elbogen et al., 2020; Brouzos et al., 2021). A psychological crisis refers to an individual encountering an event or situation that has a significant psychological impact and cannot effectively cope with it using existing psychological resources. It may cause an imbalance or dangerous state in cognition, emotion, body, or behavior (Everly and Mitchell, 2000). The occurrence of major emergencies, including COVID-19, whose transmission methods are complicated and fast-changing, has caused panic and powerlessness among the public to a large extent. The mental health of the public has been greatly affected by psychological crises or even psychological diseases emerging in some areas (Tang et al., 2020; Abir et al., 2021).

**Figure 1:**
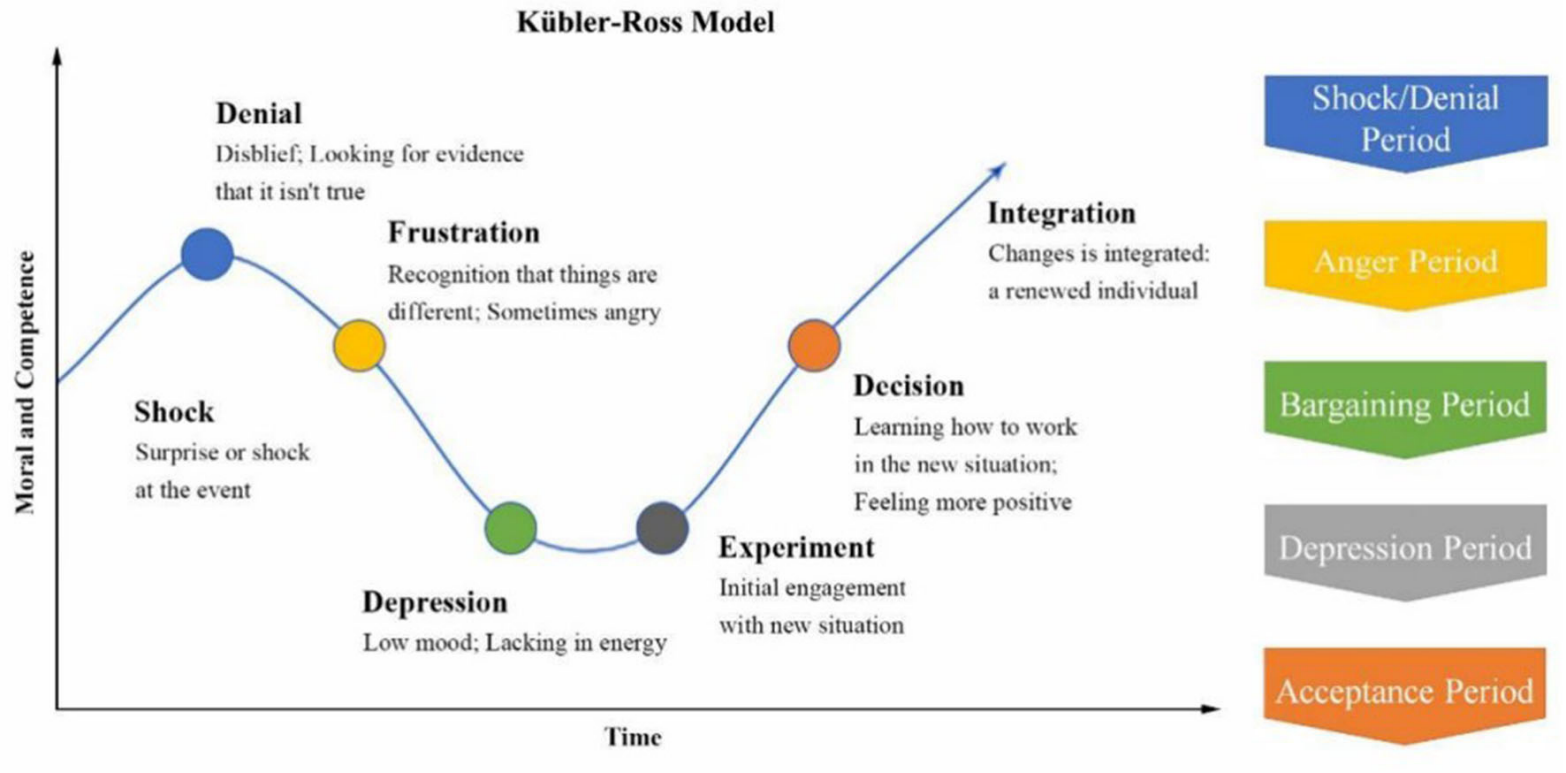
Kubler–Ross change curve.

Kubler–Ross has proposed the Kubler–Ross Change Curve which explains people's psychological changes in the face of major changes. As shown in Figure 1, Kubler–Ross Change Curve has divided people's psychological changes after encountering major changes into five periods. The periods include shock/denial period, anger period, bargaining period, depression period, and acceptance period. Following the Kubler–Ross Change Curve, we divide psychological crisis intervention under the COVID-19 pandemic into three stages: the emotional holding stage, the mental health repair stage, and the future development education stage.

### Psychological Crisis Intervention

Psychological crisis intervention refers to assisting the individual or groups who suffered from a psychological crisis in short term, thus preventing or reducing the potential negative effects of psychological trauma on the individual under the guidance of psychological theory (Everly and Boyle, 1999). The widespread applications of modern group psychological counseling started with the counseling of post-war soldiers during World War II (Terr, 1992). Group counseling is widely used in psychological counseling for children and youth groups who have experienced disasters such as earthquakes and hurricanes (Grolnick et al., 2018). This activity has a significant effect on post-disaster mental health (Austin and Godlesk, 1999). Small group intervention can have long-term effects or reduce either Post-traumatic stress disorder (PTSD) or acute stress disorder perse (De Gaglia, 2006; Newman et al., 2014; Fu and Underwood, 2015; Ali, 2020).

As a social species, humans require a safe and secure social environment to survive and thrive (Hawkley and Cacioppo, 2010), therefore, having access to supportive and affiliative relationships with others is crucial (Pérès et al., 2021). Evidence has shown that supportive relationships have a wide range of positive benefits. Supportive and affiliative relationships are physiologically and psychologically regulating, as they bring security to our world by activating affect systems associated with safety and soothing (Cacioppo et al., 2015).

Compared with other emergencies, besides panic, anxiety, fear, etc., the major lifestyle changes brought by COVID-19 is that the public must wear masks and isolate themselves at home to reduce infections. This has brought along interpersonal alienation and a perceived feeling of isolation, resulting in social environments characterized by a high degree of (social) threat (Cacioppo et al., 2011). Being deprived of supportive relationships due to perceived loneliness (Hawkley and Cacioppo, 2010), ostracism or social exclusion (Williams, 2007), social isolation (Cacioppo et al., 2011), and rejection (Gerber and Wheeler, 2009) are associated with negative consequences. A lack of social connectedness is even associated with increased morbidity and mortality (Shiovitz-Ezra and Ayalon, 2010), thus it compromises the integrity of physical and mental health and wellbeing (Hawkley and Cacioppo, 2010).

Facing the psychological crisis caused by COVID-19, Chinese psychologists pioneered the creation of a compound model mental health intervention system that divides objects and hierarchies, and vigorously advocated network intervention methods (Zhong et al., 2020). In particular, online group psychological intervention is critical to crisis intervention (Mitchell et al., 2009; Brouzos et al., 2021). Telemental health using network or other technological options has been suggested as a practical and efficient alternative for psychological crisis intervention (Holmes et al., 2020; Brouzos et al., 2021), especially for those who are more vulnerable to COVID-19 (Courtet et al., 2020).

In conclusion, although some existing literature have studied the problem of online psychological intervention during COVID-19, the scope of the population involved is small and it is still in the experimental stage, whose timeliness is poor for the sudden and fast-spreading COVID-19. Therefore, we established and launched an online intervention model which covered the whole country and multiple groups such as medical staff and police.

## Study Design

### Online Working Platform and Staff

Because we were all locked down at home, so we established an online working platform and organized a staff team for this program, by combining the WJX (a platform that can easily design questionnaires and collect data), Official WeChat, WeChat group, and the Zoom. In this way, we could stay at home and recruit staff members, train group leaders, recruit members, do online group counseling, and collect feedback, all could be done *via* the internet.

A brochure was published by the Official Wechat, which was that any individual who felt stressed during this pandemic can apply for this service, they can click the QR code on the brochure and fill out an application form. After submitting the application form, they will be redirected to scan another QR code which will bring them into a WeChat group, where they can get information about how to receive OESAG service, they will get another QR which will collect their feedback after the group session.

To do this program, we need different people to do different tasks, we first listed the tasks, such as drafting a recruitment brochure, inserting the application form into WJX and creating a QR code, inserting the QR code into the brochure, publishing the brochure on the official WeChat to recruit group members, design training course to train qualified group leaders, do the administrative job for group members and group leaders, design questionnaires and analyze the data, write an academic report, etc.

People should first confirm that they are not in an active psychosis state before they apply.

**Figure 2:**
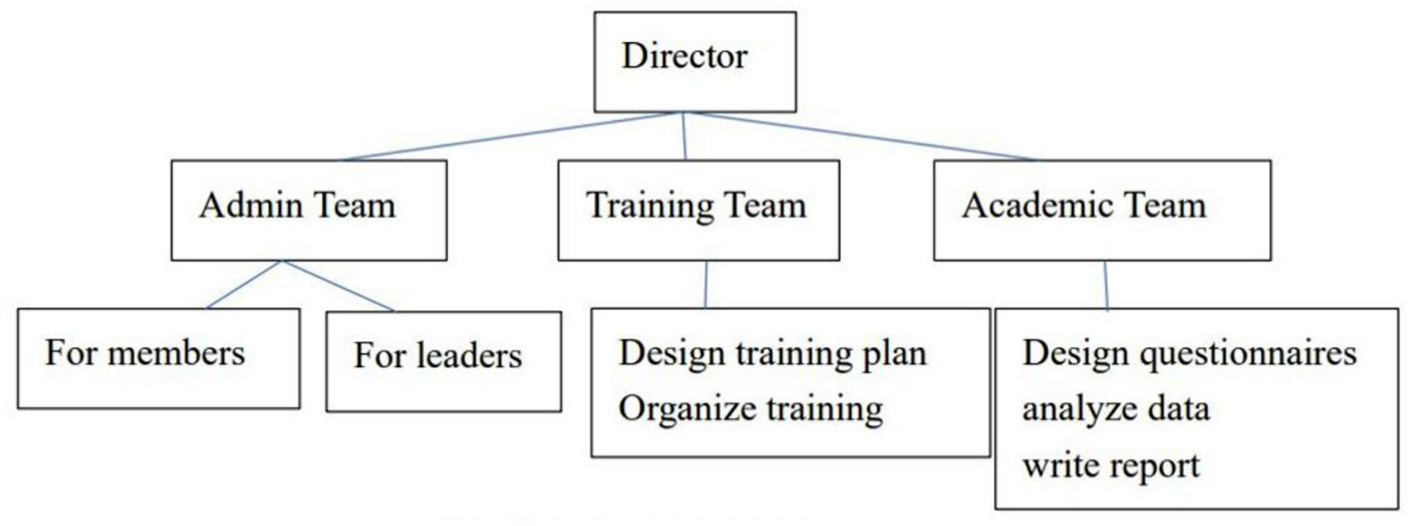
Staff team structure.

We structured the staff team according to the tasks as Figure 2. Members referred to those who participated in the OESAG. Leaders referred to the group counselors, whom we call group leaders.

### Measures

We designed the members' application form, member feedback questionnaire, and a group-leader feedback questionnaire. The application form was used to analyze the emotional change of the public during COVID-19, and the feedback form of members and leaders was used to help us explore the effectiveness of OESAG from two perspectives of members and leaders, respectively.

#### Members Application Form

This form asks for the member's name, gender, age, occupation, location, and what brings him/her to this group. The applications are collected before they enter the group.

#### Member Feedback Questionnaire

The feedback questionnaire is designed around emotional expressions, mutual support, subjective stress state, etc. It includes certain questions like members' goal achievement, gains, what's helpful, future expectations, and more. The feedback are collected after each group.

#### Group-Leader Feedback Questionnaire

The feedback questionnaire is designed around group effectiveness (emotional linkage, resource extension, listening, security, emotional catharsis, emotional transition) and group leaders' intervention. It includes questions such as what did you do during this group, what are the issues that appeared in the group, how is the focus member, and how do you think the goal achievement? The feedback are collected after each group.

### Procedure

OESAG is designed to provide a space for members to share their emotions and get support from each other. It is meant to help members transform their emotions and get the power to face uncertainty and helplessness as well as regain control. This group model, innovated by us, lasts for 60 min comprised of the following four to five steps:

(1) Group leaders introduce themselves in a sentence, introduce the setting of the group, including the purpose of the group, what can (and cannot) be provided by the group, the issue of confidentiality, and will ask for members' thoughts. A secure group climate with clear boundaries can thus be set up in this way. This last for about 10 min.
(2) A meditation exercise such as meditation music or relaxation to relax members to cross the boundary from daily life into the group. This step is optional. A group leader can decide whether to do this according to the group dynamics. This last for about 5 min.
(3) Group leaders invite members to talk about their experience during the past 1 month, especially a typical story, the story can be a family story, or workplace story, around their emotions, thoughts, behavior, interpersonal interactions, etc. Each member has 3 min to share their story in a sequence of names on the screen. This last for about 30 min. If the focus member appeared, the group leader can decide to extend this step to take care of this focus member, to avoid hurting this focus member.
(4) Group leaders invite each member to give feedback to another member on what he/she heard and appreciated, like “I heard your story, I appreciated you for your …” In this way, members help each other to find their own power. This last for about 15–20 min.
(5) Members will reflect on their experiences, and the group leaders will end the group. This last for about 10 min.

### Data and Methods

The data of this paper came from the public welfare project, “Psychological Services for Psychological Rehabilitation Groups under the COVID-19 Pandemic Situation” and has received strong support from Professor Fumin Fan of Tsinghua University and Beijing Normal University. We established OESAG to conduct psychological crisis intervention for the public during the COVID-19 pandemic. The project was conducted between February 10 to 17 (phase one), 2020, February 18 to March 1 (phase two), and March 8 to April 9 (phase three). OESAG was conducted *via* the online working platform we established. A total of 53 groups were recruited. A total of 481 persons have applied to participate, among them 440 have gotten the offer, 346 of them have participated, and 253 members along with 139 group leaders have sent their feedback. All the data are listed in Table 1.

**Table 1:** Sample and data of the OESAG.

**Data resource**	**First period (2.10–2.17)**		**Second period (2.18–2.29)**		**Third period (3.8–4.9)**	
**Number of groups**	**8**		**24**		**21**	
	**Application form**	**Feedback form**	**Application form**	**Feedback form**	**Application form**	**Feedback form**
Group members	84	54	227	119	261	80
Group leaders		9		57		73

NVivo (Version 12.0, Qualitative analysis software, QSR International, Melbourne, Australia) and SPSS (Version 22.0, IBM, USA) are used for data analysis. We analyzed the application forms at three periods to understand the emotional change of the public during the COVID-19 pandemic and analyzed the feedback forms to explore the effectiveness of OESAG by triangular mutual printing methods which included qualitative analysis, quantitative analysis, and case analysis. Specifically, we used NVivo 12 to analyze the feedback forms of group members and leaders to explore the effect of OESAG on public psychological crisis intervention by extracting high-frequency words from responses to the feedback forms of group members and leaders, used SPSS 22 to explore the reliability and validity of the feedbacks of group members and leaders, and used the case analysis to further confirm the results of the qualitative analysis and quantitative analysis.

## Results and Analysis

Using NVivo to qualitatively analyze the 572 application forms, 253 member feedback, and 139 group-leader feedbacks, we explored the evolution and characteristics of the public emotional change during the pandemic as well as the effectiveness of OESAG.

### Public Emotional Change During the Pandemic

**Figure 3:**
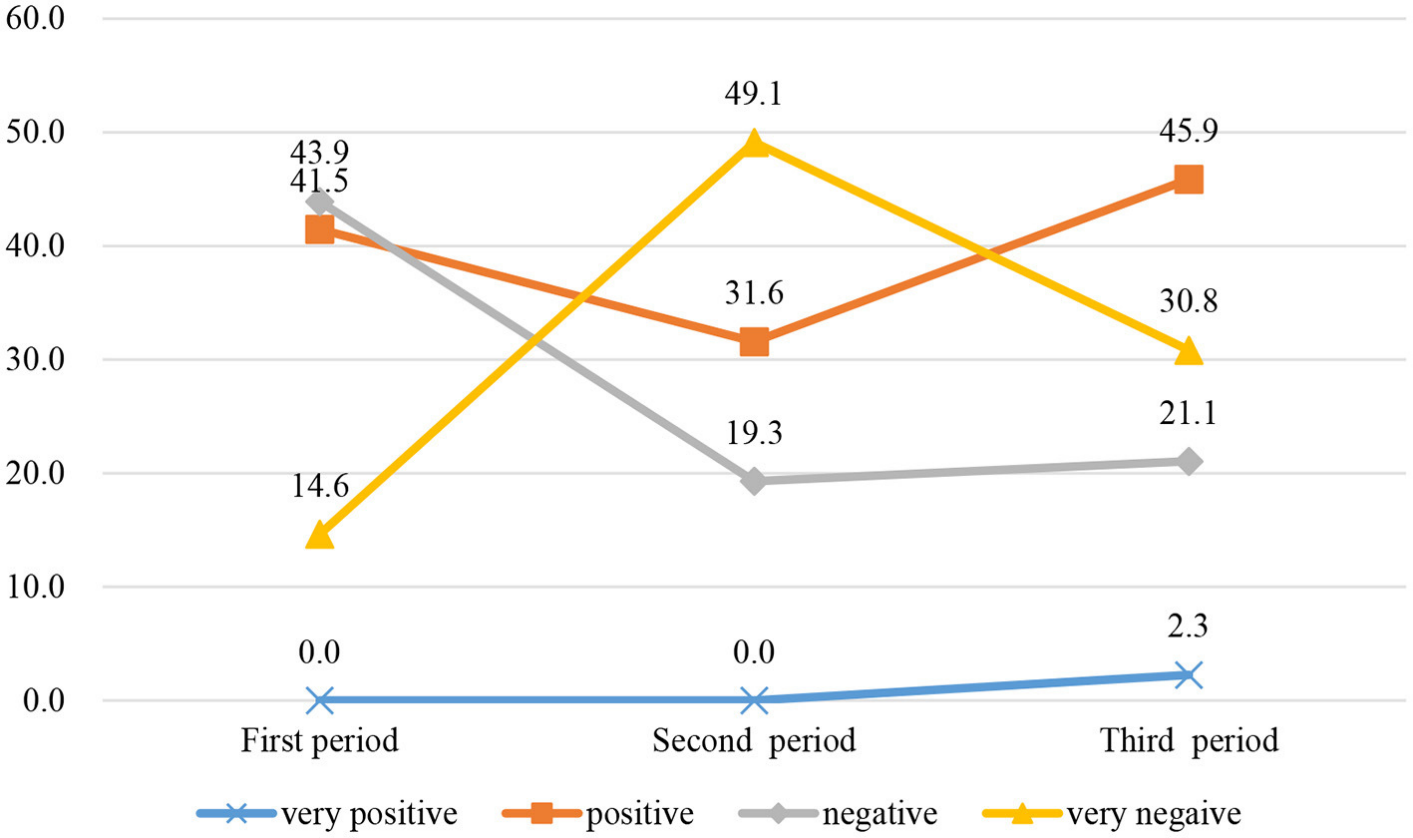
The evolution of group members' emotions in three periods.

COVID-19 rapidly spread in China in February 2020 when we established OESAG to deal with the psychological crisis caused by COVID-19. In the application form, we divide the emotions of group members into these four categories: very positive, relatively positive, relatively negative, and very negative, and the members will evaluate and choose according to their own situation. Based on the application forms submitted by group members, we have analyzed the public emotional changes during the COVID-19 pandemic. Figure 3 shows the evolution of group members' emotions in three periods. In the first period, the proportions of members with very positive, relatively positive, relatively negative, and very negative emotions were 0, 41.5, 43.9, and 14.6%, respectively. Compared with the first period, the members with very negative emotions in the second period have greatly increased. This is consistent with the Kubler–Ross change Curve. As the pandemic gradually stabilized, the life, work, and study of the member also gradually returned to normal. Compared with the second period, the number of members with positive emotions in the third period has greatly increased, and members with negative emotions have decreased.

We further analyzed the mentality trajectories of group members in the COVID-19 pandemic with an analysis of the demands of group members to participate in OESAG. Table 2 presents the demands of group members in OESAG in three periods. The recruitment time of the first and second phases is close, and the members' demands for the group were relatively similar. The members had more needs for “support,” “company,” “anxiety,” “relieve,” etc. The demands of the members in the first period focused on the work stress caused by the pandemic, so they were more willing to establish connections with others in hopes of obtaining company and psychological support from OESAG. OESAG can help members relieve anxiety and provide professional psychological support advice. In the second period, “support” and “company” were still the main demands of the members, and their anxiety had increased significantly. Compared with the first period, members in the second period mentioned “understand” and “listen,” expressing their desire to have listened to and to relieve anxiety through OESAG. As the pandemic gradually stabilized, the demands of the prospective members changed significantly in the third period, and “work” began to become the main topic of concern for the members again. The recovery of online work and part of offline work increased the sense of reality for members and their work stress caused by the pandemic was alleviated. As the public gradually returned to normal work or looked for new jobs, the demands for “psychological support,” “relieve stress or anxiety,” and “emotional adjustment” were apparent.

**Table 2:** The demands of group members at three periods.

**Order**	**First period**			**Second period**			**Third period**		
	**Word**	**Frequency**	**Weighted percentage**	**Word**	**Frequency**	**Weighted percentage**	**Word**	**Frequency**	**Weighted percentage**
1	Support	25	5.05	Support	35	5.58	Psychology	32	2.53
2	Company	14	3.37	Company	25	3.99	Support	39	2.53
3	Psychology	11	2.64	Anxiety	22	3.51	Work	31	2.45
4	Anxiety	11	2.64	Relieve	18	2.87	Study	28	2.22
5	Relieve	11	2.64	Psychology	15	2.39	Relieve	25	1.98
6	Development	12	2.52	Study	13	2.07	Stress	24	1.90
7	Pandemic	10	2.40	Experience	13	1.99	Pandemic	24	1.90
8	Work	9	2.16	Understand	13	1.75	Development	34	1.87
9	Stress	6	1.44	Listen	10	1.59	Anxiety	22	1.74
10	Professional	5	1.20	Pandemic	9	1.44	Emotional adjustment	22	1.74

In conclusion, the evolution of group members' emotions and the changes in the demands of group members during the three periods indicated the importance of prompt intervention in the first period of the pandemic.

### Effectiveness of OESAG

#### Qualitative Analysis

**Figure 4:**
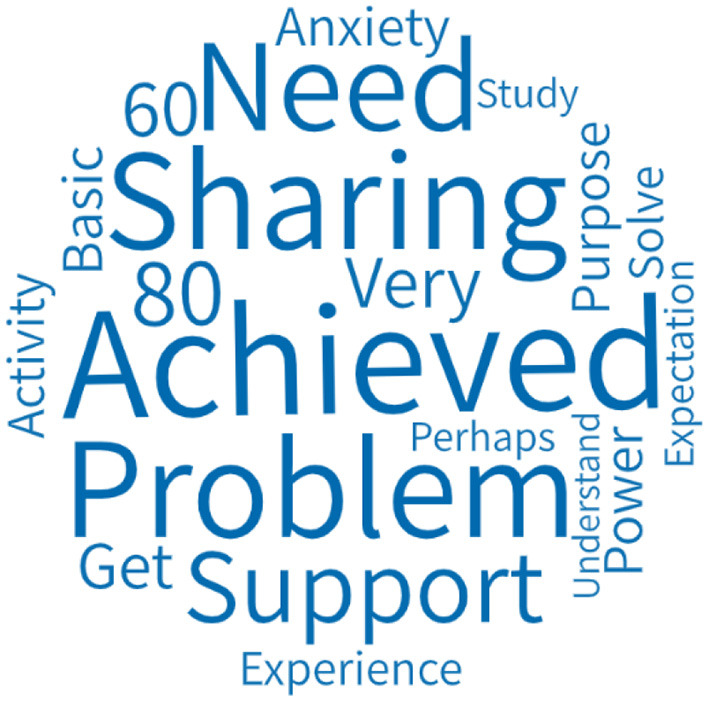
Goal achievement evaluation of OESAG.

We analyzed 253 members' feedback forms over three periods from the aspect of goal achievement evaluation of OESAG. Figure 4 shows the word cloud of the goal achievement evaluation of OESAG. The key words are achieved, sharing, problem, support, need, etc. Most group members believe that OESAG's expected goals are basically achieved, and the completion degree is between 60 and 80%. They think that their emotions have been vented and they have found strength by helping each other, listening, and communicating with the leadership of group leaders. However, because of the lack of time and other factors, the achievement was not 100%.

We analyzed the feedback forms from 130 leaders of the latter two periods from four aspects. They include themes of OESAG, group dynamics of OESAG, the psychological crisis of focus members, and goal achievement evaluation of OESAG. The leaders' feedback form in the first period is relatively simple and is different from that of the latter two periods, so we did not conduct a word cloud analysis on the leaders' feedback for the first period.

**Figure 5:**
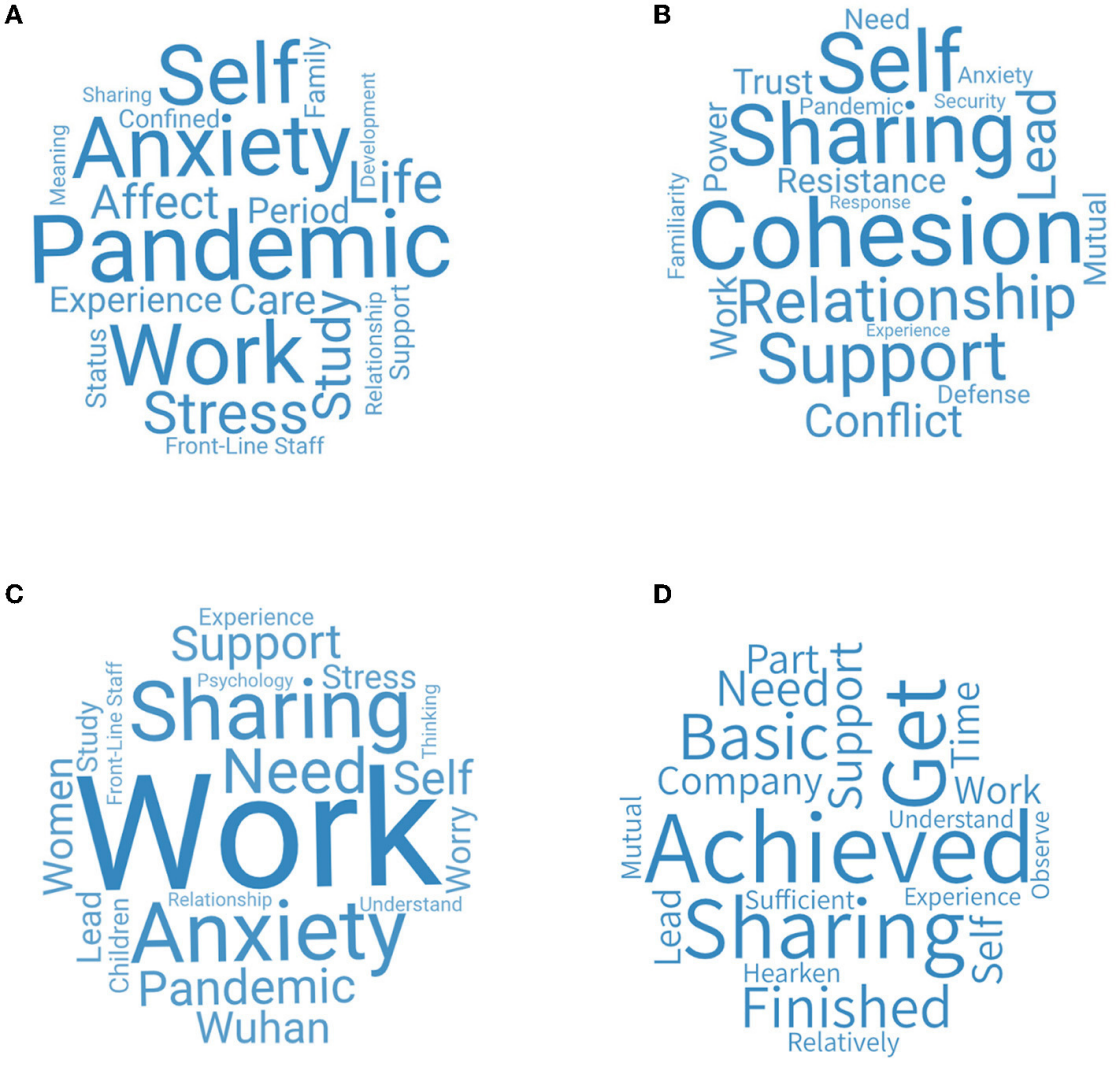
The feedback of group leaders. **(A)** Themes of OESAG. **(B)** Group dynamics of OESAG. **(C)** Psychological crisis of focus members. **(D)** Goal achievement evaluation of OESAG.

Figure 5A shows the word cloud figure of the themes of OESAG. The key words for OESAG's themes are pandemic, anxiety, self, stress, study, life, etc. Since people can only stay at home due to the pandemic, there were anxiety and pressure on their lives, studies, and work. On the one hand, people are threatened by the pandemic and had a sense of panic; on the other hand, because of the pandemic, they had to stay at home, which was inconvenient to their daily life, study, work, etc. Some people may even lose their jobs. Therefore, they needed a platform to talk and vent their emotions so that they can receive emotional support from others.

Figure 5B presents the word cloud figure about the group dynamics of OESAG. The main words are cohesion, relationship, support, sharing, conflict, etc. During the pandemic, people stayed at home and faced conflicts in various aspects such as family relations, study, work, etc. By guiding the group members to share their own experiences and emotions, the leaders gave them a sense of security in the group to break through the psychological defense and resistance between members, thus establishing a relationship of mutual trust, safety, and stability. Furthermore, the group was able to form a cohesive force and strong group dynamics to ensure the achievement of group goals.

Figure 5C shows the word cloud figure of the psychological crisis of focus members. The key words are work, anxiety, sharing, pandemic, support, women, etc. Leaders also focused on describing the focus members, especially female focus members, who suffered a greater psychological impact from the outbreak as well as higher levels of stress, anxiety, and depression (Wang et al., 2020). They found that the pandemic not only brought anxiety to members' work or study, but also great challenges to front-line staff (such as teachers, police, community workers, psychological counselors, and civil servants) whose emotions are also in need of attention (Zhang et al., 2021).

Figure 5D presents the goal achievement evaluation of OESAG. The key words are achieved, support, company, sharing and get, etc. Most leaders believed that the goals of OESAG have basically been achieved. Group members had enough time to speak and share their own feelings and experiences, thus a link through mutual listening, companionship, and support was formed to relieve the tension caused by the pandemic. However, there are also a few leaders who thought that OESAG had some problems. The problems include lack of group dynamics, not enough participating members, and separation of individual members. These are aspects that we need to think through and improve on in the future.

#### Quantitative Analysis

In order to prove the effectiveness of OESAG, we conducted a reliability and validity analysis of the feedback of group members and leaders on goal achievement in three periods. Corresponding questions about the goal achievement received in OESAG are set in the feedback questionnaires of group members (the emotional link between members, resources presented by the group, etc.) and leaders (the degree of goal achievement, emotions have been vented, etc.). The specific questions are tabulated in Tables 3, 4. And the options are very incompatible, incompatible, and very incompatible, and members or leaders can choose the corresponding options according to their conditions.

**Table 3:** Reliability and validity analysis of members' goal achievement.

**Items**	**Reliability**		**Validity**		
	**Single**	**Total**	**KMO**	**Bartlett**	***p***
Emotional link between members	0.856	0.880	0.830	299.246	0.000
Resources presented by the group	0.864				
Members' listening to each other	0.870				
Members' sense of security in the group	0.871				
Emotional venting of group members	0.850				
Emotional transformation of group members	0.842				

**Table 4:** Reliability and validity analysis of leaders' goal achievement.

**Items**	**Reliability**		**Validity**		
	**Single**	**Total**	**KMO**	**Bartlett**	***p***
The degree of goal achievement	0.738	0.744	0.702	429.910	0.000
Emotions have been vented	0.741				
Pressure has been reduced	0.733				
Feel no longer alone	0.731				
Get the company and support of your peers	0.724				
Regain control	0.744				
Instill good hope	0.726				
Group atmosphere helps relieve stress	0.726				
Groups help to channel emotions	0.720				
Get the company and support of peers in the group	0.728				
Establish good interpersonal links	0.722				
Help build confidence	0.713				
Full of hope	0.717				

Table 3 shows the reliability and validity analysis of members' goal achievement in the group. Each item's coefficients of Cronbach's alpha are all above 0.8, and the total coefficient of Cronbach's alpha is 0.880, which is also above 0.8, indicating that the reliability effect is very good. In addition, the Kaiser–Meyer–Olkin (KMO) is 0.830 > 0.6 (Kaiser and Rice, 1974), and the significance level of Bartlett's test of sphericity is Sig = 0.000 < 0.050, indicating that the significance test (Pett et al., 2004) was passed and the validity was proven to be good. In summary, the good reliability and validity once again prove that members had basically achieved their goals. Members have received support and interpersonal links, their stress has been relieved, and their emotions have been vented through OESAG.

Table 4 shows the reliability and validity analysis of leaders' goal achievement in the group. Each item's coefficients of Cronbach's alpha are all above 0.7, and the total coefficient of Cronbach's alpha is 0.744 which is also above 0.7, indicating that the reliability effect is quite good. In addition, the Kaiser–Meyer–Olkin (KMO) is 0.702 > 0.6 (Kaiser and Rice, 1974), and the significance level of Bartlett's test of sphericity is Sig = 0.000 < 0.050, indicating that the significance test was passed (Pett et al., 2004) and the validity is good. In conclusion, the good reliability and validity once again prove that the leaders had basically achieved their goals in OESAG.

In addition, due to the difference in the content of the feedback questionnaire for the third and first two periods, we analyzed the feedback form for the third period separately. Based on the data of the third period, we used SPSS to analyze the group effectiveness evaluation of the group members and leaders to further verify the effectiveness of OESAG.

Table 5 shows the effectiveness evaluation of 80 group members on OESAG. The items of effectiveness evaluation include: “I was very close to other members in the group,” “I had new discoveries and learning in the group,” “I was being listened to carefully by other members in the group,” “I felt very safe in the group,” “My emotions in the group were vented,” “I became more positive after joining the group”. The ratio of group members who believed that these items were “consistent to very consistent” is 66.25, 90, 92.5, 82.5, 86.25, and 88.75%. The remaining members believed that these items were either “not sure, inconsistent, or very consistent,” which is a minority. Therefore, we can see that most of the group members had a positive evaluation of OESAG and believed that they have gained a lot from OESAG.

**Table 5:** Effectiveness evaluation of members on OESAG (*N* = 80).

**Items**	**Evaluation**				
	**Very inconsistent**	**Inconsistent**	**Not sure**	**Consistent**	**Very consistent**
I was very close to other members in the group	4 (5%)	11 (13.75%)	12 (15%)	36 (45%)	17 (21.25%)
I had new discoveries and learning in the group	3 (3.75%)	2 (2.5%)	3 (3.75%)	41 (51.25%)	31 (38.75%)
I was listened carefully by other members in the group	3 (3.75%)	2 (2.5%)	1 (1.25%)	39 (48.75%)	35 (43.75%)
I felt very safe in the group	4 (5%)	4 (5%)	6 (7.5%)	34 (42.5%)	32 (40%)
My emotions in the group were vented	3 (3.75%)	4 (5%)	4 (5%)	47 (58.75%)	22 (27.5%)
I became more positive after joining the group	4 (5%)	1 (1.25%)	4 (5%)	47 (58.75%)	24 (30%)

Table 6 shows the effectiveness evaluation of 36 group leaders on OESAG. The items of effectiveness evaluation include: “Members had new discoveries and learning in the group,” “Members listened carefully to each other,” “Members felt very safe in the group,” “The emotions of the members in the group were vented,” and “Members became more positive”. The ratio of group members who believed these items were “consistent to very consistent” is 94.45, 100, 91.67, 97.22, and 91.66%, which are all above 90%. These data indicate that most of the group leaders have a positive evaluation of OESAG and believe that OESAG has a positive effect on group members by effectively addressing their psychological crisis of members.

**Table 6:** Effectiveness evaluation of leaders on OESAG (*N* = 36).

**Items**	**Evaluation**				
	**Very inconsistent**	**Inconsistent**	**Not sure**	**Consistent**	**Very consistent**
Members had new discoveries and learning in the group	0 (0%)	2 (5.56%)	0 (0%)	20 (55.56%)	14 (38.89%)
Members listened carefully to each other	0 (0%)	0 (0%)	0 (0%)	19 (52.78%)	17 (47.22%)
Members felt very safe in the group	0 (0%)	1 (2.78%)	2 (5.56%)	20 (55.56%)	13 (36.11%)
The emotions of the members in the group were vented	0 (0%)	0 (0%)	1 (2.78%)	23 (63.89%)	12 (33.33%)
Members became more positive	0 (0%)	1 (2.78%)	2 (5.56%)	17 (47.22%)	16 (44.44%)

#### Case Analysis

After analyzing the reliability and validity of the feedback from group members and leaders, we used the cases of our program to further confirm the effectiveness of OESAG on public psychological crisis intervention.

In the first and second periods, the purpose and appeals of group member recruitment are mostly brief. They include “reducing anxiety and easing emotions” (Case 01SD-XMY), “can be listened to and supported, and reap personal growth” (02GD-YLC). In contrast, the personal appeals of the members of the third period are more complete, and it is easier for the leader to discover them as the focus members. We conducted a case analysis of member 03BJ-XHJ in the third period.

The personal appeal described by member 03BJ-XHJ before entering the group is described as:

“*In interpersonal relationships, when you feel your own bad emotions, such as getting along with people you don't like, being hurt by language, being snatched away by others, and facing them. In this situation, how can I protect myself from harm. Leave no psychological shadow on myself and help myself grow.” (Case 03BJ-XHJ)*

The member 03BJ-XHJ cited the influence of multiple negative situations such as “getting along with people you do not like,” “being taken away by others,” and “being hurt by language,” but you “also need to face them,” which fully manifests his/her shock, frustration, and anger. In the feedback after OESAG, the member 03BJ-XHJ felt “listening, and I felt that I was being cared for and concerned with, and this promoted the smooth progress of everyone's communication. At the same time, the leaders woke us up from asking questions about anxiety to paying attention to what should be done, which also woke me” (Case 03BJ-XHJ). This is a reflection of the process of the member being paid attention to and building trust with the group. “Pay attention to everyone and allow them to speak and maintain a good communication atmosphere” (Case 03BJ-XHJ). This engages the members to start self-exposure, and members feel that “it is very necessary for everyone to communicate together as their own problems will appear smaller in the group, and the power of the group is great” (Case 03BJ-XHJ). In addition, the member 03BJ-XHJ also found that “there is something beyond my reach. Although there is no answer yet for my own question, I was able to start thinking on my own and needs to be completed by myself” (Case 03BJ-XHJ), which is an explanation of self-effort and a manifestation of loneliness. But on the whole, this is an integrated new individual, “it can make other members speak louder and stronger” (Case 03BJ-XHJ). This shows that a powerful new individual wants to make a sound.

Moreover, the leader of the group also reported that member 03BJ-XHJ's “self-awareness” is critical. This was listed as one of the focus members. The leader believed that the group where the member 03BJ-XHJ belongs “will soon establish a mutually supportive relationship” and “shows a strong cohesion.” The sharing of the group has led to changes in the members who reacted negatively at the beginning of the group.

### Mechanism of OESAG

In response to the question “what do you think are the resources obtained during the group process,” we further analyzed the feedback of group members and leaders to explore the mechanism of OESAG to promote emotional regulation.

**Figure 6:**
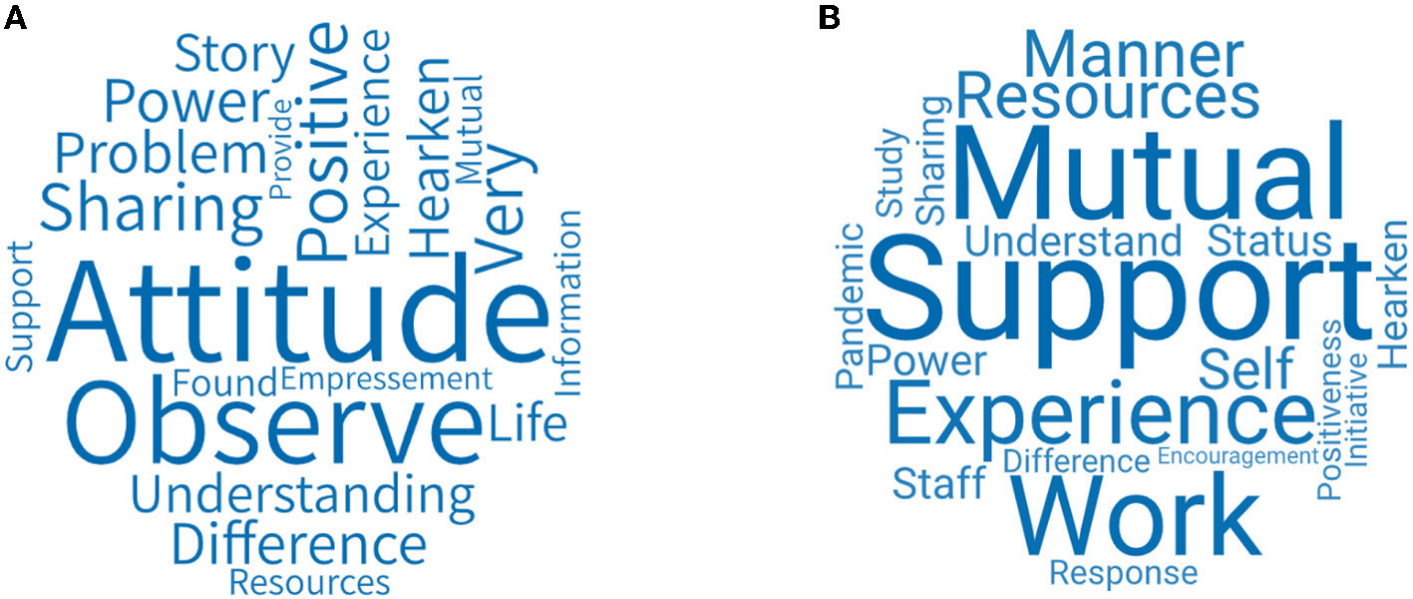
**(A)** Resources obtained in OESAG considered by group members. **(B)** Resources obtained in OESAG considered by group leaders.

Figure 6A shows the word cloud for the resources obtained in OESAG considered by the group members. The key words include attitude, positive, experience, observation and understanding, etc. The word that the members mentioned the most is “attitude”—that is, OESAG provided members with the emotions and attitudes of other members facing life and grief during the pandemic. In addition, they believe that their companions had opened up their hearts, sincerely communicated with each other and that their support for each other had also given them hope and positive strength.

Figure 6B shows the word cloud figure of the resources obtained in OESAG considered by group leaders. The key words include support, mutual, experience, sharing, work, etc. The main resource provided by OESAG is a platform for group members to establish connections with each other. Leaders guide group members of different professions to share their own stories, and to listen, support, and encourage each other. This way, they can perceive their own and others' attitudes and statuses when dealing with the pandemic. In addition, professionals will conduct further targeted assessments and provide emotional counseling services for group members.

## Discussion and Conclusions

COVID-19 has demanded unprecedented actions regarding the delivery of mental health services to the public. The provision of face-to-face group psychotherapy was unavailable because of the self-quarantine. The rapid deployment of virtual online group psychotherapy services for the public under COVID-19 stress was imperative given their level of psychiatric need.

OESAG was an effective modality of group psychotherapy under the pandemic. It can provide a secure virtual space for the public to express their anxiety, depression, fear, and disappointment. They can also get support from other members to alleviate their stress and recover from COVID-19 stress. OESAG provides a steady, convenient online communication platform for group leaders and members by combining Official WeChat, WeChat group, WJX, and Zoom. Group members can join a WeChat group by scanning the QR after they submit the application form. They can get the Zoom information in the WeChat group, then go into a Zoom room to get OESAG service. Compared with the traditional psychological crisis intervention, OESAG provides an efficient, cost-effective, viable, acceptable mental healthcare platform (Bolton and Dorstyn, 2015; Gentry et al., 2019). In addition, given that people must stay home due to COVID-19 and offline communication activities being unavailable, OESAG fully demonstrates the advantages of using modern information technologies such as WeChat, WJX, and Zoom to communicate remotely.

We first explore the mentality trajectories of the group members in the COVID-19 pandemic, then use SPSS 22.0 and NVivo 12.0 to explore the effect of OESAG on public psychological crisis intervention from two perspectives of qualitative and quantitative analysis, respectively, and finally carry out a case analysis, forming triangular cross-validation with qualitative analysis and quantitative analysis. Firstly, the mentality trajectories of members ranged from shock to depression to positive. As the pandemic gradually stabilized, “work” became the main topic of concern once gained for the public, and their stress or anxiety mainly came from returning to normal work or looking for a new job which is consistent with the Kubler-Ross Change Curve (Kubler-Ross, 1969). In addition, OESAG conducted psychological crisis interventions on group members through online methods which have effectively played a role in regulating the negative emotions of members. Due to COVID-19, people ad to stay home and maintain a social distance from others. This exacerbated their feelings of rejection, anxiety, and depression (Naqvi, 2020; Taylor et al., 2020b), which may cause psychological crisis (Abir et al., 2021; Pérès et al., 2021). OESAG provides a platform for people during the COVID-19 pandemic to share their emotions and help them feel more positive and powerful. Moreover, female groups suffered a greater psychological impact from the outbreak as well as higher levels of stress, anxiety, and depression during COVID-19 (Wang et al., 2020). COVID-19 also brought great challenges to frontline staff such as medical staff. At the initial stage of the pandemic, the government had focused more on the mental health of patients and neglected medical staff (Ang et al., 2013). However, compared with the general public, they have more direct and long-term contact with patients with COVID-19, exposing them to a higher risk of contracting the pandemic (Montemurro, 2020) and thus more likely to experience negative emotions (Zhang et al., 2021). Furthermore, OESAG aims to eliminate or reduce the negative emotions caused by the COVID-19 pandemic by establishing emotional links between group members. Under the leadership and self-demonstration of group leaders, group members had communicated and shared with each other to form a mutually supportive emotional link to relieve stress, anxiety, or other negative emotions of members. Therefore, policy makers should establish a systematic psychological crisis intervention system and a supportive effect evaluation system as soon as possible to give more attention to psychological crisis intervention for female and frontline staff.

This study has achieved quite much. First, we described the mentality trajectories of the public in China amid the COVID-19 pandemic and confirmed the Kubler–Ross Change Curve, which is from shock to depression to positive, and indicated the importance of prompt intervention at the beginning of the pandemic. Second, we have established a model of temporary organization for such an online program and innovated a model of online group counseling known as OESAG. This is a new internet platform to provide online group counseling that includes the WeChat official account, WeChat group, WJX software, and Zoom. Third, we examined the relationship between psychological stress alleviation and OESAG to provide big data for policy making of emergency intervention during and after the pandemic. We have also been able to provide suggestions to make up for the lack of psychological intervention and psychological assistance in the emergency management system in China.

However, OESAG also has its limitations. First, the control of time. Group members have given feedback that the communication was relatively short and it was not enough to fully share their emotions. Second, the number of members in each group should also be managed to avoid having too many or too few members in each group due to absence, late arrival, early leaving, etc. Third, the psychological crisis intervention for different groups lacks obvious pertinence. Finally, due to the sudden outbreak of the COVID-19 pandemic, the time of organizing OESAG is relatively urgent, so there is no systematization in the recruitment criteria, and everyone can apply.

Therefore, we think OESAG not only provides the function of the experiment but also provides the function of observation and treatment, so we will further establish a systematic platform so that it can be quickly activated to relieve the psychological crisis of the public after a major health incident. This way, we can help the public quickly log on to the platform and get the opportunity to share their own emotions, listen to others, as well as feel the link and support. We should begin by constructing a more structured program to better connect the various processes of the platform and train the leaders more efficiently. We should then aim to set a supportive group to provide people with space to express themselves, be listened to, and feel connected. In this way, the number of people will not be limited to just a consulting group. In addition, if there are enough psychological experts involved, we can establish a broad platform, so the intervention of large-scale government public psychological managers can be initiated in the future. Finally, we should also find ways to further improve the pertinence of psychological crisis intervention for different groups, such as frontline staff, the general public, and patients, in the future.

## Data Availability Statement

The original contributions presented in the study are included in the article/supplementary material, further inquiries can be directed to the corresponding author.

## Ethics Statement

The studies involving human participants were reviewed and approved by the Ethical Review Board of Beijing Jiaotong University. The patients/participants provided their written informed consent to participate in this study.

## Author Contributions

XL, XW, and YZ worked on the study design and method. XL was responsible for manuscript design, data analysis, and article writing while XW, YZ, ZM, and SH supervised the analysis and put forward constructive comments on the article. TB and DT provided interpretive input and language corrections. All authors contributed to this work, critically reviewed, and approved the final manuscript.

## Funding

This research was supported by Shenzhen-Hang Kong Institute of Brain Science and Beijing Jiaotong University (Grant No. NYKFT2020004&KOL21004530) under the name “Research on Psychological Service Technology of Psychological Rehabilitation Group under the COVID-19 pandemic”, and Hubei Key Laboratory of Human Development and Mental Health Central China Normal (Grant No. 2019B08) under the name “Post-Pandemic Integrative Mental Health Rehabilitation Group Intervention Research on Adolescents' Sense of Shame”. XL is the project host. The funding body did not play any role in the design of the study and in the writing the manuscript but in the collection of data and charges providing.

## Conflict of Interest

The authors declare that the research was conducted in the absence of any commercial or financial relationships that could be construed as a potential conflict of interest.

## Publisher's Note

All claims expressed in this article are solely those of the authors and do not necessarily represent those of their affiliated organizations, or those of the publisher, the editors and the reviewers. Any product that may be evaluated in this article, or claim that may be made by its manufacturer, is not guaranteed or endorsed by the publisher.
